# Interdisciplinary assessment and treatment of paediatric drooling: two decades of experience by the Nijmegen saliva control team reflected in a stepwise algorithm

**DOI:** 10.1007/s00431-024-05658-5

**Published:** 2024-06-28

**Authors:** Lynn B. Orriëns, Sandra A. F. de Groot, Jan J. W. van der Burg, Frank J. A. van den Hoogen, Karen van Hulst, Corrie E. Erasmus

**Affiliations:** 1 Department of Paediatric Neurology, Division of Paediatrics, Donders Institute for Brain, Cognition and Behaviour, Radboudumc Amalia Children’s Hospital, Radboud University Medical Center, Geert Grooteplein Zuid 10, Nijmegen, the Netherlands; 2 Department of Rehabilitation, Donders Institute for Brain, Cognition and Behaviour, Amalia Children’s Hospital, Radboud University Medical Center, Nijmegen, the Netherlands; 3 Department of Paediatric Rehabilitation, Sint Maartenskliniek, Nijmegen, the Netherlands; 4 School of Pedagogical and Educational Science, Radboud University Nijmegen, Nijmegen, the Netherlands; 5 Department of Otorhinolaryngology/Head and Neck Surgery, Radboud University Medical Center, Nijmegen, the Netherlands

**Keywords:** Saliva control team, Interdisciplinary care, Drooling, Children, Clinical decision-making

## Abstract

Anterior and posterior drooling are prevalent comorbidities in children with neurodevelopmental disabilities. Considering the heterogeneity of the patient population and the multifactorial aetiology of drooling, an interdisciplinary and individualised treatment approach is indispensable. However, no tool for stepwise decision-making in the treatment of paediatric drooling has been developed previously. Within the Radboudumc Amalia Children’s Hospital, care for children with anterior and/or posterior drooling secondary to neurodevelopmental disabilities is coordinated by a saliva control team with healthcare professionals from six disciplines. In alignment with international literature, published guidelines, and evidence gained from two decades of experience and research by our team, this paper proposes an algorithm reflecting the assessment and treatment approach applied in our clinic. First, directions are provided to decide on the necessity of saliva control treatment, taking type of drooling, the child’s age, and the severity and impact of drooling into account. Second, the algorithm offers guidance on the choice between available treatment options, highlighting the importance of accounting for child characteristics and child and caregiver preferences in clinical (shared) decision-making.

*Conclusions*: With this algorithm, we aim to emphasise the importance of repeated stepwise decision-making in the assessment and treatment of drooling in children during their childhood, encouraging healthcare professionals to apply a holistic approach.

**Table:** 

**What is Known:** • *Children with anterior or posterior drooling secondary to neurodevelopmental disabilities comprise a heterogeneous group, necessitating an individualised treatment approach.* • *No stepwise decision-making tool is available for the treatment of paediatric drooling.*
**What is New:** • *Deciding on the necessity of saliva control treatment should be a conscious process, based on type of drooling, age, and drooling severity and impact.* • *Type of drooling, age, cognition, oral motor skills, self-awareness, posture, diagnosis, and child/caregiver preferences need to be considered to decide on the optimal treatment.*

**Supplementary Information:**

The online version contains supplementary material available at 10.1007/s00431-024-05658-5.

## Introduction

Children and youth with neurodevelopmental disabilities commonly experience problems with oral motor control. Consequently, in addition to limitations in eating and drinking abilities, anterior and posterior drooling are prevalent comorbidities. Anterior drooling, defined as the unintentional loss of saliva from the mouth [1], is observed in 44% of children with cerebral palsy (CP) [2]. Posterior drooling, which is the spilling of saliva over the base of the tongue into the pharynx, leading to pooling of saliva or saliva aspiration [3, 4], is estimated to occur in 10–15% of children with severe or profound intellectual disabilities [4].

Anterior and posterior drooling each have a distinct yet profound impact on the daily lives of children and their families, resulting from physical and psychosocial sequelae and a considerable burden of care [4, 5]. Fortunately, an increasing number of treatment options have become available to diminish drooling [3]. Considering the heterogeneity of the patient population and the multifactorial aetiology of drooling, an interdisciplinary and individualised treatment approach is indispensable [1].

Internationally, an effort has been made to optimise the treatment approach for drooling, of which several examples are presented in Table 1. Nevertheless, no approach for stepwise decision-making, deciding on the optimal treatment approach while taking specific characteristics of the child into account, has been suggested previously. Building on two decades of experience in caring for this patient population, our saliva control team has established consensus on such an approach. Hence, this article aims to provide a comprehensive overview of our team’s methodology, suggesting a step-by-step approach to the assessment and treatment of anterior and posterior drooling.

**Table 1:** Examples of international efforts to optimise management of paediatric drooling

Focus	Title of publication	Year of publication
Assessment and treatment in general	Saliva control issues in the neurologically challenged. A 30-year experience in team management	2005
	American Academy for Cerebral Palsy and Developmental Medicine (AACPDM) Care Pathway for Sialorrhea in Cerebral Palsy	2017
	Impact and management of drooling in children with neurological disorders: an Italian Delphi consensus	2022
	Comprehensive management of anterior drooling: An International Pediatric Otolaryngology Group (IPOG) consensus statement	2023
Specific treatment	Botulinum toxin assessment, intervention and aftercare for paediatric and adult drooling: international consensus statement	2010
	A consensus statement on the use of botulinum toxin in paediatric patients	2021
	Botulinum neurotoxin type A in the interdisciplinary treatment of sialorrhea in adults and children – update and practice recommendations	2023
Outcome measurement	Drooling outcome measures in paediatric disability: a systematic review	2022

## Methods

Children with saliva control problems who visit the Radboudumc Amalia Children’s Hospital undergo comprehensive assessment and—when indicated—treatment by our saliva control team (Table 2). Drawing on the expertise of various disciplines, the team fosters an interdisciplinary decision-making approach, in line with the insights provided by Crysdale and colleagues [6].

**Table 2:** Composition and focus of the Nijmegen saliva control team

**Team composition**	
Involved disciplines	The clinical team spans six disciplines, comprising a paediatric neurologist, speech-language therapists, otorhinolaryngologists, a healthcare psychologist, a remedial educationalist, and (originally) a rehabilitation physician (who has now retired)
Consulted specialists	A specialised dentist, physical therapist, dietician, or gastroenterologist are consulted by the team when required
**Patient population**	
Numbers	The team has been involved in the care for approximately 1000 children over the past two decades, of whom roughly 600 received saliva control treatment. Due to growth and development, these children often required repeated assessment and treatment of their saliva control problems during childhood
Referrals	Children were referred from primary, secondary, or tertiary care centres across the Netherlands, including those outside the primary catchment area of our tertiary care hospital
Clinical characteristics	Children ranged from infants to young adults (approximately 0–18 years), with primary diagnoses including cerebral palsy, neurogenetic disorders or syndromes, as well as acquired brain injury
**Treatment methodology**	
Evidence	In addition to patient care, the team has conducted scientific research, which has contributed to the development of assessment tools, knowledge on the effectiveness and impact of available treatment options, the establishment of potential side effects, and the identification of predictive factors for treatment effect and side effects

Through ongoing multidisciplinary meetings, where new scientific evidence is analysed, insights from patient care are shared, and their implications for our treatment approach are evaluated; our team has seamlessly integrated innovative treatment options, revised assessment methods, and key factors influencing treatment outcomes in our care methodology. This iterative process has refined our methodology into an evidence- and practice-based interdisciplinary approach that centres around the child and their family. Aligned with the phases of the clinical reasoning cycle [7], this approach embodies a commitment to holistic patient care.

Informed by the collective expertise of our team members, the approach applied by our saliva control team has been distilled into a stepwise algorithm guiding the assessment and treatment of anterior and posterior drooling throughout childhood.

## Results

**Fig. 1:**
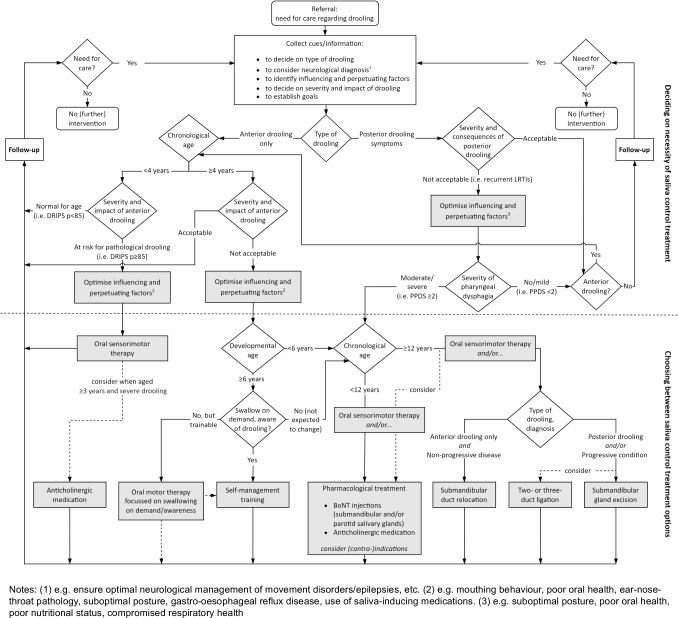
Comprehensive flowchart representing the treatment approach for children and youth with anterior or posterior drooling secondary to neurodevelopmental disabilities

The proposed algorithm for the assessment and treatment of anterior and posterior drooling in children and youth with neurodevelopmental disabilities is detailed in Fig. 1.

### Baseline assessment

Children with a need for care regarding drooling are referred to our saliva control clinic by a physician. All information guiding treatment decisions is subsequently collected during a baseline assessment when the child, together with their parent(s)/caregiver(s) (referred to as ‘caregivers’ throughout this paper), visits our clinic. The assessment comprises three sequential components.

First, a medical assessment, an evaluation of the child’s learning abilities, self-management skills and social functioning (e.g. interaction with social environment, self-awareness, and response to negative reactions on drooling), and an oral motor assessment are conducted, in line with recommendations [1, 3]. Through these assessments, it is deduced whether the child has anterior drooling, posterior drooling, or both [8], and factors that influence and perpetuate drooling are identified. For example, anterior drooling may be influenced by frequent mouthing behaviour, poor oral health, suboptimal posture, and ear-nose-throat pathology [9]. Additionally, gastro-oesophageal reflux disease or medication use may contribute to excessive salivary flow [8]. In children with posterior drooling, the condition may be negatively influenced by—among other things—a reclined sitting position, poor oral health, compromised respiratory health, and dysmotility of the gastrointestinal tract [4]. This approach is rooted in considering the autonomic nervous system, including the neurological pathways that influence saliva production [8].

Second, the severity, frequency, and impact of drooling are quantified. Anterior drooling is assessed through a combination of (semi-)objective and caregiver-reported measures [3], including the drooling quotient, a verbal numerical rating scale reflecting drooling severity, and the Drooling Severity and Frequency Scale (DSFS). Furthermore, the impact of drooling in daily life is discussed and quantified [5]. This discussion includes an inventory of the child’s social context at home and at school, to estimate the extent to which drooling affects social interaction and well-being, and the extent to which the child and parents/caregivers are able to follow through with recommendations. Caregiver-involvement in these assessments (and child-involvement, if possible) is considered essential, as they hold the key to understanding the true extent of the severity and impact of drooling in daily life. For children up to 4 years old, the Drooling Infants and Pre-schoolers Scale (DRIPS) is administered to quantify the child’s severity and frequency of drooling relative to their typically developing peers.

For posterior drooling, clinical history taking is used to assess saliva aspiration risk, for example with regard to repeated episodes of aspiration pneumonia and need for antibiotics, need for suctioning, deteriorating pulmonary condition, and choking incidents [3]. While no recommendations are available on the (non-invasive) quantification of posterior drooling [3], the assessment in our centre is supplemented by caregiver-reported severity of posterior drooling symptoms and cervical auscultation to assess the pharyngeal phase of swallowing. The presence and severity of posterior drooling can subsequently be quantified using the Paediatric Posterior Drooling Scale (PPDS), a 5-point classification ranging from clear to wet breathing before and after swallowing.

Third, treatment goals are established and child and caregiver preferences for treatment are identified. As a shared decision-making process between caregivers and healthcare professionals, it is discussed whether these goals are attainable. If possible—taking age, cognition, self-awareness and communicative abilities into account—the child is directly involved in this process. Otherwise, caregivers are considered advocates for their child.

### Treatment approach

During an interdisciplinary consultation, the saliva control team discusses and interprets all information collected at the baseline assessment to reach consensus on a recommended treatment approach. This decision-making process consists of three phases, which are summarised in Table 3 and explained in detail in the Appendix.

First, the team determines whether it is necessary to initiate saliva control treatment, which is generally based on three main characteristics: (1) the type of drooling, (2) the (chronological) age of the child, and (3) the severity, frequency, and impact of drooling.

Second, if saliva control treatment is indicated, the team decides on the most suitable treatment option. This may involve improving the child’s saliva management (e.g. oral sensorimotor training, self-management training), reducing the volume of saliva (e.g. pharmacological treatment, salivary duct ligation, or submandibular gland excision) or rerouting the salivary flow (e.g. submandibular duct relocation). Regardless of which treatment option is decided on, our team recommends intermittent oral sensorimotor therapy as an add-on treatment.

In a third phase, comprehensive (telephone) follow-up is ensured, either to evaluate effectiveness and potential side effects of treatment, or to re-evaluate the need for care and provide additional advice when no saliva control treatment was initiated.

**Table 3:** Key contributors to the decision-making process for management of anterior and posterior drooling across three different phases

**Phase 1: Deciding on the necessity of initiating saliva control treatment**	
a) Why is it important to differentiate between anterior and posterior drooling?	Given the potentially life-threatening nature of posterior drooling, its management should take priority over anterior drooling, irrespective of the child’s age. Additionally, some treatment options are not suitable for children with posterior drooling, which should be considered in the decision-making process
b) How do we determine whether anterior drooling is pathological?	Anterior drooling is considered pathological in children aged 4 years or older. For those under four, DRIPS percentile scores are assessed to indicate whether they are at risk for pathological drooling compared to typically developing peers. Information on drooling severity, frequency, and its impact on daily life is used determine the acceptability of drooling in children aged four years and older, in a shared decision with the child and caregivers
c) Why should influencing and perpetuating factors be considered?	Addressing influencing and perpetuating factors that were identified during the assessment (e.g. prescribing anti-reflux medication in case of anterior drooling) may already diminish drooling and eliminate the necessity for further treatment
**Phase 2: Deciding on the most suitable treatment option**	
a) Why is it important to consider developmental age?	Children with an estimated developmental age of at least 6 years may be taught a self-management routine in order to achieve saliva control. In addition to this treatment being less (medically) invasive than other options, children will develop internal control of saliva instead of applying symptom management through saliva reduction
b) How do we choose between treatment options that reduce saliva volume or reroute salivary flow?	As saliva control may still improve over time, pharmacological treatments to reduce saliva volume are preferred over surgical options up to the age of 12 years. When considering surgical treatments, factors that may affect eligibility (e.g. duct relocation is contraindicated in children with pharyngeal dysphagia) or outcomes are carefully evaluated. Child and caregiver preferences are key factors in this decision-making process. For example, pharmacological treatment may still be an option for older children if they or their caregivers do not wish to proceed to more invasive surgical procedures
**Phase 3: Scheduling follow-up appointments**	
a) When is follow-up implemented?	Standardised (telephone) follow-up consultations are scheduled after all pharmacological and surgical treatments, both in the short term (8 weeks) and longer term (32 weeks), to assess treatment effectiveness, discuss experiences and satisfaction, and identify a new or ongoing care needs. Additionally, trimonthly telephone evaluations are implemented for children on anticholinergic medication to ensure the continued appropriateness of the treatment

These phases are explained in detail in the Appendix

*DRIPS* Drooling Infants and Pre-schoolers Scale

## Discussion

This paper presents the interdisciplinary approach to assessing and treating drooling in children and youth with neurodevelopmental disabilities as implemented by our saliva control team. Our clinical reasoning-based algorithm provides a detailed, stepwise decision-making tool for healthcare professionals involved in the care of children with anterior and/or posterior drooling. Crucially, the algorithm is designed to be dynamic, evolving with ongoing research and new treatment advancements to ensure optimal, personalised care.

The developed algorithm underscores two critical contributors to the decision-making process. First, a thorough baseline assessment is paramount in enabling informed decision-making at each decision node and devising individual care plans, attentive to the needs, values, and preferences of children and their families. Second, an interdisciplinary approach that brings together the expertise of various specialists is essential in gathering diverse information and weighing the pros and cons of different treatment options.

The decision-making process is split into two phases, for which the algorithm provides child characteristics that should be accounted for. First of all, initiating saliva control treatment requires careful consideration and should not be an automatic decision upon referral to a saliva control clinic. In our experience, refraining from active intervention may sometimes be the best approach. Meanwhile, clinicians involved in the child’s care team have the potential to contribute to optimised saliva control by evaluating and addressing influencing and perpetuating factors. This proactive approach may obviate the need for referral to a saliva control team. Second, the choice between the available treatment options should be based on characteristics that affect the invasiveness of considered treatments (i.e. the child’s age and type of drooling), determine their feasibility (i.e. developmental age, oral motor skills, and awareness of drooling), or influence effectiveness or safety (i.e. posture, type of drooling, and diagnosis). Available literature commonly states that less invasive interventions should be preferred over more invasive interventions [10], yet accounting for these characteristics enables a child-centred treatment approach in which non-invasive interventions (e.g. oral sensorimotor therapy, self-management training, or anticholinergic medication) may not only precede but also follow after invasive interventions or may be omitted altogether (e.g. when a child first visits the saliva control team at age 12 or older and has a developmental age below 6 years, surgical treatment may be the most suitable choice right away).

Finally, follow-up assessment is considered an important addition to the algorithm. In terms of determining treatment effectiveness, the international literature has gradually shifted away from only using objective measures towards incorporating caregiver-reported measures and metrics that quantify the impact of drooling on activities and participation. As illustrated by Rosenbaum, “we have become increasingly aware of the importance of patients’ (and families’) voices” [11]. Likewise, our team’s approach evolved from determining treatment effectiveness based on pathophysiologic parameters (e.g. salivary flow rate and drooling quotient), to additionally using caregiver-reported measures (e.g. verbal numerical rating scales and DSFS) and quantifying differences in the impact of drooling on daily life (e.g. using a caregiver-reported questionnaire). Eventually, we aim to implement an additional individualised outcome measure that involves identifying specific situations where drooling has a major impact and rating saliva control in those situations, facilitating follow-up assessments focused on aspects that are most important to each child and family. The psychometric properties of this measure are currently being studied.

A strength of the presented algorithm is that, besides being evidence-based, it incorporates practice-based evidence accumulated through 20 years of experience by our saliva control team. In addition, effective clinical reasoning is known to enhance the quality of patient care and improve patient outcomes [7]. Applying this approach will enable healthcare professionals to focus on the child, engaging in cue-based decision-making, and providing child-centred care by tailoring treatment to fit their unique needs.

Nevertheless, it is important to acknowledge the limitations of the algorithm. First, the decision nodes in the algorithm reflect the treatment approach applied by our team. Although the approach is rooted in international literature, there are grey areas where alternative choices may be valid. For example, treatments that may be available elsewhere (e.g. medical taping, oral appliances, tactile cueing, salivary gland ablation) are not included. Moreover, some treatments included in the algorithm may not be universally accessible. Nevertheless, by classifying available treatments into one of the aforementioned treatment groups (i.e. improving saliva management, reducing saliva volume, rerouting salivary flow), the algorithm provides valuable insight into the characteristics essential for child-centred decision-making even in these cases. Second, when applying a child and family-centred approach, it is essential to have flexibility to tailor the approach to the specific needs of the child and their family. We sought to integrate this flexibility into the current algorithm as much as possible, but clinicians must consider whether the suggested paths align with the individual needs of each child or whether a different approach may be more appropriate in specific cases.

In summary, this paper introduces a stepwise algorithm for assessing and treating drooling in children with neurodevelopmental disabilities. We aim to inspire healthcare professionals to adopt a holistic approach that considers each child’s unique characteristics and social context to guide decision-making. Our intention is to spark global dialogue and collaboration among saliva control teams, fostering the exchange of best practices, beginning with the publication of our own treatment methodology and its rationale, and ultimately enhancing clinical care.

### Supplementary Information

Below is the link to the electronic supplementary material.

Supplementary file1 (DOCX 45 KB)

## Data Availability

No datasets were generated or analysed during the current study.
