# Supplementary material for: Interdisciplinary assessment and treatment of paediatric drooling: two decades of experience by the Nijmegen saliva control team reflected in a stepwise algorithm
Source: Eur J Pediatr. 2024 Jun 28;183(9):3979–85. doi: 10.1007/s00431-024-05658-5 (PMC11322295; doi:10.1007/s00431-024-05658-5)
Supplement: Supplementary file 1 — Supplementary file1 (DOCX 45 KB) [file 431_2024_5658_MOESM1_ESM.docx]

**APPENDIX**

| This Appendix serves as a supplement to the paper titled “*Interdisciplinary Assessment and Treatment of Paediatric Drooling: Two Decades of Experience by the Nijmegen Saliva Control Team Reflected in a Stepwise Algorithm*”. It provides a detailed description of the three decision-making phases in the proposed treatment approach for anterior and posterior drooling in children with neurodevelopmental disabilities, expanding upon the brief overview provided in the main paper. |
| --- |

**Phase 1: Is it necessary to initiate saliva control treatment?**

The necessity of saliva control treatment is generally determined based on three main characteristics: 1) the type of drooling, 2) the (chronological) age of the child, and 3) the severity, frequency and impact of drooling. Additionally, it is assessed whether factors that influence and perpetuate drooling can be addressed, potentially precluding the need for further treatment.

*a) Why is it important to differentiate between anterior and posterior drooling?*

Due to the potential risk of saliva aspiration, which may be life threatening [1], posterior drooling requires attention irrespective of the child’s age, prioritizing its management over anterior drooling. Only when symptoms are mild and consequences are deemed acceptable, follow-up and reassessment may suffice. Additionally, it is essential to consider that certain treatment options are not suitable for children with posterior drooling, influencing the decision-making process.

*b) How do we determine whether anterior drooling is pathological?*

Anterior drooling is generally considered pathological in children aged four years or older [2]. The criteria to determine the acceptability of anterior drooling, therefore, depend on the child’s (chronological) age.

For children under four, Drooling Infants and Pre-Schoolers Scale (DRIPS) percentile scores from situation- and sex-specific reference charts will help decide whether saliva control is abnormal compared to typically developing peers [3]. Monitoring by a primary care-based speech-language therapist (adapted to the individual child and their caregivers) in anticipation of maturation and improvement of saliva control is recommended for children with scores below the 85^th^ percentile, as this indicates that drooling may not be significantly more than peers [4]. Sometimes, general advice is given to optimise the situation. A follow-up assessment is typically conducted after three months, involving a re-evaluation of the DRIPS and an assessment of the current need for care. In children with scores above the 85^th^ percentile, indicating that they are at risk for pathological drooling, oral sensorimotor therapy is advised. Based on the situations addressed in the DRIPS, individualised recommendations regarding improving saliva control can be provided.

For children aged four years and older, the acceptability of drooling is determined based on information collected about the severity and impact of drooling in daily life. Instead of using specific cut-off values, this is a shared decision with children and caregivers.

*c) Why is it important to consider influencing and perpetuating factors?*

Addressing influencing and perpetuating factors that were identified during the assessment may already diminish drooling and eliminate the necessity for further (saliva control) treatment. For example, in the case of anterior drooling, anti-reflux medication might be prescribed to manage gastro-oesophageal reflux disease [5], dental treatment may be recommended, or treatment of allergies or chronic rhinitis may be advised. For children with posterior drooling, interventions such as using a positive expiratory pressure (PEP) device to optimise airway clearance [6] or prescribing prokinetics to improve gastric motility may be suggested [7].

**Phase 2: Which treatment option is most suitable?**

When choosing to initiate saliva control treatment, treatment may either concern improving the child’s saliva management (e.g. oral sensorimotor training, self-management training), reducing the volume of saliva (e.g. pharmacological treatment, salivary duct ligation, or submandibular gland excision) or rerouting the salivary flow (e.g. submandibular duct relocation). Several demographic and clinical characteristics of the child can be used to guide decision-making.

*a) Why is it important to consider developmental age?*

Children with sufficient cognitive abilities may be trained to perform a self-management routine to achieve saliva control. In addition to this treatment being less (medically) invasive than other options, children will develop internal control of saliva instead of applying symptom management through saliva reduction [8]. Children with an estimated developmental age above 6 years are generally eligible for this treatment option [8-10], considering the growing self-conscience and self-reflection of children from this age. Before inclusion, it is important to consider their ability to swallow on demand (as established during oral motor assessment [11]), their awareness of drooling and its practical and social consequences, and their (intrinsic) motivation to improve saliva control. These are necessary characteristics for the treatment to be successful [8]. Moreover, a significant facilitator that enhances treatment success – and one that should be considered before treatment – is the extent to which the child’s social environment is prepared to support the child in learning and consistently applying these self-management skills.

*b) How do we choose between treatment options that reduce saliva volume or reroute salivary flow?*

In children with neurodevelopmental disabilities, whose development may be more gradual than typically developing children, saliva control might still improve over time. This is why our team prefers pharmacological treatment to reduce saliva volume over surgical treatment options up to the age of 12 years [12-14]. Our clinical experience has shown that pharmacological treatment will generally control drooling sufficiently up until this age. For children with posterior drooling, surgical approaches may be initiated at a younger age when saliva poses a serious threat to pulmonary health. Contra-indications are considered when choosing between systemic (i.e. anticholinergic medication) or localized (i.e. botulinum neurotoxin type A injections) pharmacological treatment options.

To inform the choice between surgical treatment options, characteristics that may affect the outcome of treatment are taken into account. Importantly, duct relocation is contra-indicated in children with pharyngeal dysphagia [15]. Children with symptoms of posterior drooling are therefore not eligible for this treatment, as are children with neurodegenerative disabilities, considering that disease progression might cause a deterioration in pharyngeal swallowing function over time. As adequate stability of the head was shown to be predictive of response to submandibular duct relocation [14], children with suboptimal posture might benefit more from submandibular gland excision, which may be considered.

Finally, child and caregiver preferences are key factors in the decision-making process. For instance, pharmacological treatment may still be considered in older children if the child or caregivers do not wish to proceed to more invasive surgical options. Moreover, if anticholinergic medication and botulinum neurotoxin A injections are both indicated and suitable, caregiver preferences will be the deciding factor.

Regardless of which treatment option is decided on, our team recommends intermittent oral sensorimotor therapy as an add-on treatment. From clinical experience it can be established that the effectiveness of treatment may be improved if it is not a stand-alone procedure.

**Phase 3: When is follow-up implemented?**

It is highly important to monitor the effectiveness and potential side effects of treatment [11]. Standardised (telephone) follow-up consults, both in the short term (8 weeks) and longer term (32 weeks), are therefore scheduled after all pharmacological and surgical treatment options. During these consults, treatment effectiveness is assessed, the experiences and satisfaction of children and caregivers is discussed, and any new or ongoing care needs are discussed. Outcome measures reflecting the severity and frequency of drooling (i.e. drooling quotient [16], verbal numerical rating scale for drooling severity, and Drooling Severity and Frequency Scale [17] for anterior drooling; verbal numerical rating scale for severity of symptoms and Paediatric Posterior Drooling Scale [18] for posterior drooling) are used to quantify differences in drooling relative to baseline. When follow-up has been concluded after surgical treatment, caregivers are actively encouraged to reach out to the team if they wish to discuss any changes in their child’s saliva control over time.

Additionally, regular telephone evaluations are implemented for children using anticholinergic medication, at least once every three months, to assess whether the treatment is still appropriate for the child. In some cases, healthcare professionals from the child’s care team (e.g. primary or secondary care-based speech-language therapists, rehabilitation physicians, paediatricians) are involved during follow-up to allow for (additional) monitoring of the child closer to home.

Moreover, even when no saliva control treatment has been initiated, our team considers it important to suggest at least a one-time follow-up assessment after several months. This facilitates a re-evaluation of the need for care and the provision of additional advice when needed.

**References**

1. Gibson N, Blackmore AM, Chang AB, Cooper MS, Jaffe A, Kong WR, et al. Prevention and management of respiratory disease in young people with cerebral palsy: consensus statement. Developmental Medicine & Child Neurology. 2021;63(2):172-82. <https://doi.org/10.1111/dmcn.14640>

2. Meningaud JP, Pitak-Arnnop P, Chikhani L, Bertrand JC. Drooling of saliva: a review of the etiology and management options. Oral Surgery, Oral Medicine, Oral Pathology and Oral Radiology. 2006;101(1):48-57. <https://doi.org/10.1016/j.tripleo.2005.08.018>

3. van Hulst K, van den Engel-Hoek L, Geurts ACH, Jongerius PH, van der Burg JJW, Feuth T, et al. Development of the Drooling Infants and Preschoolers Scale (DRIPS) and reference charts for monitoring saliva control in children aged 0-4 years. Infant Behavior & Development. 2018;50:247-56. <https://doi.org/10.1016/j.infbeh.2018.01.004>

4. Challenges in managing drooling in children. Drug and Therapeutics Bulletin. 2015;53(6):66-8. <https://doi.org/10.1136/dtb.2015.6.0331>

5. Walshe M, Smith M, Pennington L. Interventions for drooling in children with cerebral palsy. Cochrane Database of Systematic Reviews. 2012(2):Cd008624. <https://doi.org/10.1002/14651858.CD008624.pub2>

6. Lagerkvist AL, Sten G, Westerberg B, Ericsson-Sagsjö A, Bjure J. Positive expiratory pressure (PEP) treatment in children with multiple severe disabilities. Acta Paediatrica. 2005;94(5):538-42. <https://doi.org/10.1111/j.1651-2227.2005.tb01935.x>

7. Curry JI, Lander TD, Stringer MD. Review article: erythromycin as a prokinetic agent in infants and children. Alimentary Pharmacology & Therapeutics. 2001;15(5):595-603. <https://doi.org/10.1046/j.1365-2036.2001.00964.x>

8. de Bruijn TWP, Sohier J, van der Burg JJW. Outpatient treatment based on self-management strategies for chronic drooling in two children. Journal of Developmental and Physical Disabilities. 2017;29(5):735-55. <https://doi.org/10.1007/s10882-017-9553-1>

9. van der Burg JJW, Didden R, Engbers N, Jongerius PH, Rotteveel JJ. Self-management treatment of drooling: a case series. Journal of Behavior Therapy and Experimental Psychiatry. 2009;40(1):106-19. <https://doi.org/10.1016/j.jbtep.2008.05.001>

10. van der Burg JJW, Sohier J, Jongerius PH. Generalization and maintenance of a self-management program for drooling in children with neurodevelopmental disabilities: A second case series. Developmental Neurorehabilitation. 2018;21(1):13-22. <https://doi.org/10.1080/17518423.2016.1232763>

11. Glader L, Delsing C, Hughes A, Parr J, Pennington L, Reddihough D, et al. AACPDM Care Pathway for sialorrhea in Cerebral Palsy 2017 [updated June 2018. Available from: <http://www.aacpdm.org/publications/care-pathways/sialorrhea>.

12. Scheffer ART, Erasmus CE, van Hulst K, van Limbeek J, Rotteveel JJ, Jongerius PH, van den Hoogen FJA. Botulinum toxin versus submandibular duct relocation for severe drooling. Developmental Medicine & Child Neurology. 2010;52(11):1038-42. <https://doi.org/10.1111/j.1469-8749.2010.03713.x>

13. Delsing CPA, Cillessen E, Scheffer ART, van Hulst K, Erasmus CE, van den Hoogen FJA. Bilateral submandibular gland excision for drooling: Our experience in twenty-six children and adolescents. Clinical Otolaryngology. 2015;40(3):285-90. <https://doi.org/10.1111/coa.12375>

14. Kok SE, Erasmus CE, Scheffer ART, van Hulst K, Rovers MM, van den Hoogen FJA. Effectiveness of submandibular duct relocation in 91 children with excessive drooling: A prospective cohort study. Clinical Otolaryngology. 2018;43(6):1471-7. <https://doi.org/10.1111/coa.13188>

15. Lawrence R, Bateman N. Surgical management of the drooling child. Current Otorhinolaryngology Reports. 2018;6(1):99-106. <https://doi.org/10.1007/s40136-018-0188-2>

16. van Hulst K, Lindeboom R, van der Burg J, Jongerius P. Accurate assessment of drooling severity with the 5-minute drooling quotient in children with developmental disabilities. Developmental Medicine & Child Neurology. 2012;54(12):1121-6. <https://doi.org/10.1111/j.1469-8749.2012.04428.x>

17. Thomas-Stonell N, Greenberg J. Three treatment approaches and clinical factors in the reduction of drooling. Dysphagia. 1988;3(2):73-8.

18. van Hulst K, Lagarde MLJ, de Groot SAF, Erasmus CE, van Den Engel-Hoek L. Reliability and validity of the pediatric posterior drooling scale: A pilot study. Dysphagia. 2017;32(1):126-207. <https://doi.org/10.1007/s00455-016-9766-y>
